# The Protective Potential of *Petroselinum crispum* (Mill.) Fuss. on Paracetamol-Induced Hepatio-Renal Toxicity and Antiproteinuric Effect: A Biochemical, Hematological, and Histopathological Study

**DOI:** 10.3390/medicina59101814

**Published:** 2023-10-12

**Authors:** Ghizlane Nouioura, Tayeb Kettani, Meryem Tourabi, Layla Tahiri Elousrouti, Omkulthom Al kamaly, Samar Zuhair Alshawwa, Abdelaaty A. Shahat, Abdulsalam Alhalmi, Badiaa Lyoussi, Elhoussine Derwich

**Affiliations:** 1 Laboratory of Natural Substances, Pharmacology, Environment, Modeling, Health and Quality of Life (SNAMOPEQ), Faculty of Sciences Dhar El-Mehraz, Sidi Mohamed Ben Abdellah University, Fez 30000, Morocco; meryem.tourabi@usmba.ac.ma (M.T.); lyoussi@gmail.com (B.L.); elhoussinederwich@yahoo.fr (E.D.); 2 Saâda Laboratory of Medical Analysis, Fez 30000, Morocco; kettanit@yahoo.fr; 3 Departments of Pathology, University Hospital Hassan II, Fez 30050, Morocco; layla.tahirielousrouti@usmba.ac.ma; 4 Department of Pharmaceutical Sciences, College of Pharmacy, Princess Nourah bint Abdulrahman University, P.O. Box 84428, Riyadh 11671, Saudi Arabia; omalkmali@pnu.edu.sa (O.A.k.); szashawwa@pnu.edu.sa (S.Z.A.); 5 Department of Pharmacognosy, College of Pharmacy King Saud University, P.O. Box 2457, Riyadh 11451, Saudi Arabia; ashahat@ksu.edu.sa; 6 Department of Pharmaceutics, School of Pharmaceutical Education and Research, Jamia Hamdard, New Delhi 110062, India; 7 Unity of GC/MS and GC, City of Innovation, Sidi Mohamed Ben Abdellah University, Fez 30000, Morocco

**Keywords:** *Petroselinum crispum*, paracetamol, hepato-renal damage, proteinuria, hematological toxicity

## Abstract

*Background and Objectives:* Paracetamol overdose is a significant global issue due to its widespread use, which can lead to a lack of awareness regarding its potential side effects. Paracetamol can harm the liver, possibly resulting in liver failure. Conversely, this study employed extracts from *Petroselinum crispum* (PC), known for its rich content of bioactive compounds, with demonstrated antioxidant properties shown in previous research as well as protective effects against various diseases. The primary objective of this study was to investigate the potential protective effects of *Petroselinum crispum* on altered hematological and biochemical parameters in the blood of rats exposed to paracetamol. *Materials and Methods:* The study involved twenty Wistar rats divided into four groups. Different groups of male rats were administered PC extract at 200 mg/kg body weight daily for 15 days, along with a standard reference dose of paracetamol at 200 mg/kg. The study assessed hepatoprotection capacity by analyzing liver enzymes such as aspartate aminotransferase (AST), alanine aminotransferase (ALT), bilirubin, albumin, and lipid profiles. Renal safety was evaluated through creatinine, urea, uric acid, lactate dehydrogenase (LDH), and total protein. Additionally, histopathological examinations of the liver and kidneys were conducted. *Results:* Following Paracetamol overdose, there were reductions in hemoglobin levels, serum total protein, albumin, and uric acid. Paracetamol overdose also elevated levels of several blood biomarkers, including creatinine, urea, nitrogen, ALT, AST, triglycerides, LDH activity, white blood cell count, and platelet count compared to the control group. However, using an ethanolic extract of *Petroselinum crispum* significantly mitigated the severity of these alterations and the extent of the effect correlated with the dose administered. Parsley extract helped prevent proteinuria and low hemoglobin, which are common side effects of Paracetamol. *Conclusions:* Therefore, parsley may hold promise in managing liver and kidney conditions—particularly in addressing proteinuria. Ultimately, these results may have implications for human health by potentially mitigating paracetamol-induced renal, hepatic, and hematological toxicity.

## 1. Introduction

The liver contributes significantly to increasing and eliminating drugs from the human body [1]. The metabolism of drugs and xenobiotics into non-toxic substances in the liver by enzymes is essential for the proper functioning of the organism [2]; the alteration of these conditions leads to a displacement towards the production of oxidants, which adhere to lipids or nuclear proteins, resulting in mutations, membrane damage, and altered enzyme activity, leading to organ dysfunction [3,4].

Paracetamol (4’-hydroxyacetanilide, N-acetylp-aminophenol, acetaminophen, PAR) is one of the most commonly used drugs, employed as an analgesic and antipyretic; it is a structural analog of phenacetin [5]. Paracetamol (=APAP) poisoning is a considerably common cause of severe acute hepatitis [6]. However, for the past four decades, paracetamol treatment nomograms have been performed to determine whether or not acetylcysteine therapy is required after an acute overdose of Paracetamol [7]. Acetylcysteine increases the risk of liver injury in a dose-dependent manner [8,9]. Furthermore, Paracetamol enters the enterohepatic circulation after absorption into the intestine and liver via its glucuronidation and sulfation, and only a tiny amount of the drug is eliminated by the kidneys [10]. At a therapeutic dose, 90% of it is eliminated by the urinary route after glucuronoconjugation and sulphuronoconjugation at the level of the hepatocytes, and 2.7 h is the mean half-life elimination [11]. The remaining 10% is metabolized by the alternative Cytochrome P450 pathway to N-acetyl p-benzoquinone imine (NAPQI) [8]. Oxidative metabolism transforms Paracetamol into its toxic metabolite (NAPQI) via the Cytochrome P450 enzyme (CYP450) [12]. There are many isoforms of CYP450: CYP2E1, CYP1A2, CYP 3A4, CYP 2D6, and CYP 2A6 [13].

**Figure 1:**
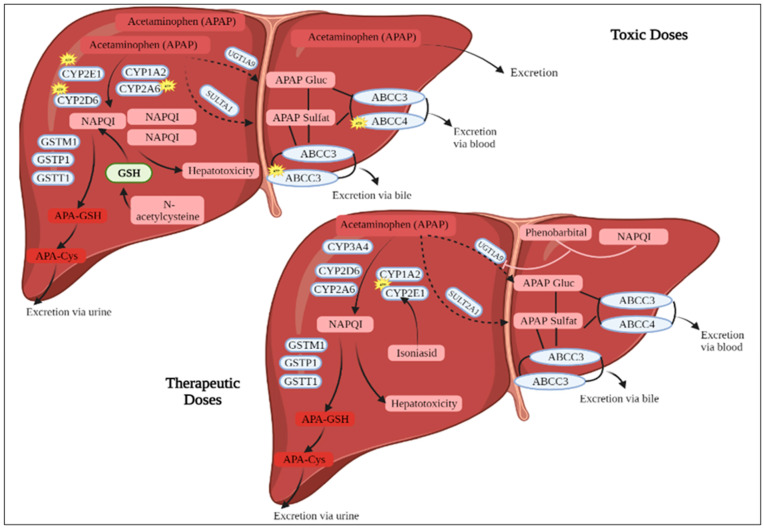
Metabolism of Paracetamol. APAP: Paracetamol, CYP2A6: Cytochrome P450 2A6, CYP2D: cytochrome P450 2D, GSH: Glutathione, GSTM1: Glutathione S-transferase Mu 1, GSTP: Glutathione S-transferase pi gene, GSTT 1: Glutathione-S-transferase theta 1, NAPQI: N-acetyl-p-benzoquinone imine.

These highly hepatotoxic compounds are then rapidly detoxified by binding to reduced glutathione (via Glutathione-S-Transferase = GST) and then eliminated in the urine after conjugation with cysteine or mercapturic acid [14]. During an overdose, the mechanisms of detoxification by conjugation are overwhelmed, and the Paracetamol is mainly metabolized by the alternative route of Cytochrome P450 to NAPQI [15]. This massive production exceeds the elimination capacity by fixing glutathione via the GST [16]. There is, therefore, an accumulation of intrahepatocyte NAPQI, which binds to cytosolic proteins and leads to mitochondrial toxicity [17]. On the histological level, cell necrosis of the Centro-hepatocyte type appears, and on the clinical level, the signs of acute hepatitis occur. Many enzymes are involved in the metabolism of Paracetamol [18]. Genetic polymorphisms (SNP = single nucleotide polymorphism) or gene copy number repeats (CNV = copy number variation) could be responsible for variability in individuals’ sensitivity to Paracetamol, which induces the development of acute hepatitis at therapeutic doses or the presence of non-serious acute hepatitis at toxic doses (Figure 1) [19]. Glucuronide conjugation is the primary way of metabolizing Paracetamol; the transformation of Paracetamol into glucuronoconjugated Paracetamol (eliminable through the urine >> bile) [20]. The enzyme responsible for glucuronoconjugation is uridine 5′-diphospho-glucuronosyltransferase (UGT) [5]. There are many isoforms, but five are particularly more involved in the metabolism of Paracetamol: UGT1A1, UGT1A9, UGT1A6, and UGTB15 [21].

Many commercially available polyherbal formulas in Morocco claim to have hepatoprotective effects [22]. The chemical components of liver-protective herbal medicines include phenols, coumarins, lignans, essential oils, monoterpenes, carotenoids, glycosides, flavonoids, organic acids, lipids, alkaloids, and xanthines [23]. In addition, plant extracts from several crude drugs are also used to treat liver disorders [24].

Parsley (*Petroselinum crispum*) is a biennial herb from the Apiaceae family [25]. It has been cultivated worldwide for centuries for food flavoring, essential oil applications, and traditional medicines [26]. Is a rich source of natural antioxidants [27]. Numerous pharmacological effects have been attributed to parsley, including those of an antioxidant, antibiotic, antifungal, neuroprotective, nephroprotective, anti-diabetic, analgesic, spasmolytic, anti-platelet, laxative, and diuretic substance [22,28,29,30,31,32].

Despite considerable advances in modern medicine, no drugs expand liver function, rescue the liver from damage, or help regenerate liver cells. Furthermore, a scientific and methodical research has still yet to be reported in the literature regarding Parsley’s action on the liver and kidney. For these reasons, the current study has been undertaken to examine the possible protective effects of *Petroselinum crispum* on altered hematological, biochemical, and oxidative stress parameters in the blood of paracetamol-treated rats to transmit the results for human use.

## 2. Materials and Methods

### 2.1. Plant Material

Fresh plants (Aerial part) of *Petroselinum crispum* (Mill.) Fuss. were planted during June–October in the flowering period in the Sefrou area. The plants were all collected at one time for to avoid any potential variations. The plants were identified taxonomically, and their voucher specimen (RAB40104) was kept in the Laboratory of Natural Substances, Pharmacology, Environment, Modeling, Health, and Quality of Life (SNAMOPEQ), Faculty of Sciences Dhar El-Mehraz, Sidi Mohamed Ben Abdellah University. The Aerial part of the Parsley was mechanically fragmented for later storage in sealed container bags at 0 °C after being dried at ambient temperature for seven days, in an airy environment away from light.

### 2.2. Preparation of Plant Extract

The World Health Organization (WHO) extraction procedure was adopted for this study. The extraction process was carried out by mixing 10 g of powder with 100 mL of hydroethanolic solvent 70% *v*/*v*; the obtained extract was centrifuged, filtered, and stored in the refrigerator at 20 °C until used.

### 2.3. In Vivo Hepatorenal Protective Activity

#### 2.3.1. Test Animals

Albino rats (200–250 g) were maintained under control conditions in the animal house of the Faculty of Science, Sidi Mohamed Ben Abdellah University, Fez. Rats were acclimatized in a room at 25 ± 1 °C, with a photoperiod of 12 h (12 L/12 D), and provided tap water and food ad-libitum. The procedures used to carry out this study were approved by our institutional committee on animal protection, as per the guidelines of the ethical approval registered under the number L.20. USMBA-SNAMOPEQ 2020-03.

#### 2.3.2. Experimental Design

Animals were divided into four groups, comprising five rats in each group.

Group 1 (control) received only 10 mL/kg of distilled water for 15 days.

Group 2 (Paracetamol-control) received 200 mg/kg daily by gavage for 15 days.

Group 3 (Normal group) received a hydro-ethanolic extract of Parsley at 200 mg/kg daily by gavage for 15 days.

Group 4 (Paracetamol + PC-Ext) received a single dose of paracetamol (200 mg/kg) + 200 mg/kg of hydro-ethanolic extract daily by gavage for 15 days. The plant extract was administered three hours after the administration of the Paracetamol.

The dose of Paracetamol and the Parsley extract was 200 mg/kg, following studies by El Menyiy et al. [6] and Jean Jacques et al. [33], respectively.

The treatments were continued for seven days, and on the 8th day of the experiment, all animals were anesthetized and sacrificed, and vital organs (liver and kidneys) were secluded and preserved in 10% formalin for histological analysis.

#### 2.3.3. Urine Collection and Analysis

The animals were maintained in metabolic cages individually to collect 24 h urine samples on days 0, 7, and 15 of treatment. Urine volume was counted directly after collection. Urine samples collected on day 15 were stored at −20 °C to determine different parameters. Urine was analyzed for protein, creatinine, urea, and uric acid.

#### 2.3.4. Blood Sampling

After 15 days of the experiment, the retro-orbital puncture collected blood samples from the anesthetized animals in all groups. Creatinine, blood urea nitrogen (BUN), uric acid, protein, and albumin levels were measured in the blood. In addition, the hepatic function was evaluated by measuring serum aspartate aminotransferase (AST), alanine aminotransferase (ALT), alkaline phosphatase (ALP), and lactate dehydrogenase (LDH).

Red blood cells (RBC), white blood cells (WBC), platelets (PLT), hemoglobin (Hb), and hematocrit (Ht) values were determined using conventional techniques on an automated hematology analyzer.

### 2.4. Statistical Analysis

Data were entered into an Excel file (Microsoft Office 2016) and analyzed with GraphPad Prism version 8.0 for Windows software. All results are expressed as mean value ± standard error of the mean (S.E.M.). Two-way ANOVAs followed by Dunnett’s multiple comparisons tests were used to statistically compare the groups. Results were considered significant at *p* < 0.05.

## 3. Results

### 3.1. Effect of PC-Ext and Paracetamol on Kidneys and Liver Function Tests

The impacts of the interventions on serum levels of albumin, creatinine, blood urea, uric acid, and total protein are illustrated in Table 1. A significant rise in creatinine, BUN, and triglycerides following a 200 mg/kg paracetamol overdose indicated kidney damage. Additionally, the uric acid level was significantly reduced compared to the control. However, the co-administration of the hydroethanolic extract of parsley (200 mg/kg) significantly inhibited the elevation of blood urea, creatinine, and triglycerides caused by the high dose of Paracetamol.

Regarding the liver function test, Paracetamol overdose markedly increased serum hepatic enzyme levels (ALT, AST, ALP, and LDH). It significantly reduced serum proteins and albumin in the control groups (Table 1). Regardless, oral *Petroselinum crispum* significantly attenuated the normalized elevated liver enzyme level.

### 3.2. Effect of Paracetamol and PC-Ext on Hematological Parameters

The results of the hematological analysis in the control and treatment groups are illustrated in Table 2. The results revealed that Paracetamol significantly reduced red blood cells, hemoglobin, hematocrit, VGM, TCMH, and CCMH. However, it induced an elevation of white blood cells, and platelets compared to the control group. The co-administration of PC-Ext with Paracetamol reversed these changes and significantly attenuated the toxic impacts of the Paracetamol.

### 3.3. Influence of Paracetamol and Parsley on Proteinuria

In Paracetamol-treated rats (Table 3), treatment induced a significant elevation of urine volume on days 7 and 15 as compared to the baseline and the control (*p* < 0.05). On day 15, urine volume was 6.3 ± 0.5 mL/day in the control group and 8 ± 1.2 mL/day in the Paracetamol group.

The urine pH, also mentioned in Table 3, in the Paracetamol-treated groups increased significantly (*p* > 0.05) compared to the control and normal groups; effects in the Paracetamol and PC-Ext treated rats were mitigated by the concomitant use of parsley.

**Figure 2:**
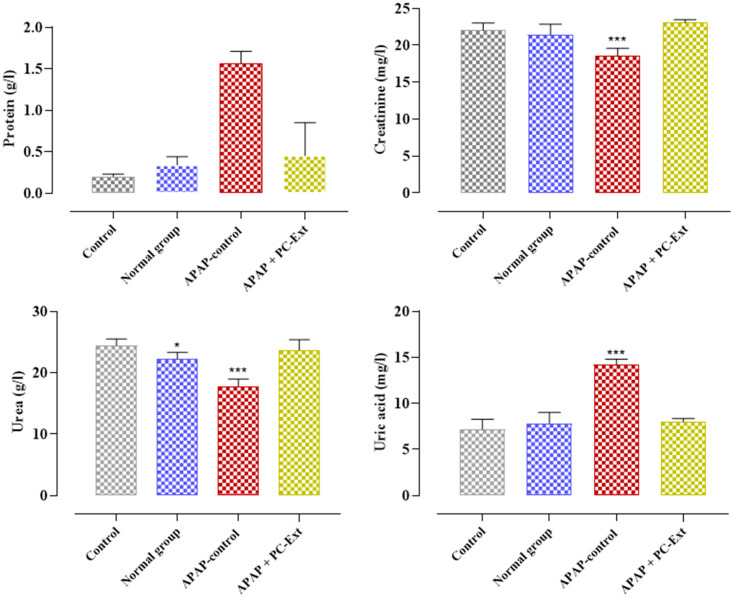
Effects of Paracetamol and PC-Ext on 24 h urinary excretion of total protein, creatinine, urea, and uric acid on day 15 after commencement of the intervention. Each value represents mean ± S.E.M. * *p* < 0.05, *** *p* < 0.001 as compared with the control group. Statistical analysis by two-way ANOVA followed by Dunnett’s multiple comparisons tests.

The effects of interventions on the 24 h urinary excretion of total protein, creatinine, urea, and uric acid on day 15 after the start of the intervention were recapitulated in Figure 2. Paracetamol significantly increased the urinary excretion of uric acid (14.23 ± 0.56 mg/L) compared to the control (7.17 ± 1.1 mg/L), whereas parsley reduced urinary uric acid. The outcomes revealed that Paracetamol overdose induced a significant decrease in the urinary excretion of creatinine (18.56 ± 1.04 mg/L) and urea (17.76 ± 1.23 mg/L), while it significantly increased the urinary excretion of proteins (1.57 ± 0.14 mg/L) as compared to the control. Regardless, the Parsley extract diminished the urinary excretion of proteins compared to the control groups and increased the urinary excretion of creatinine and urea compared to the Paracetamol-control groups (Figure 2).

### 3.4. Histological Findings

**Figure 3:**
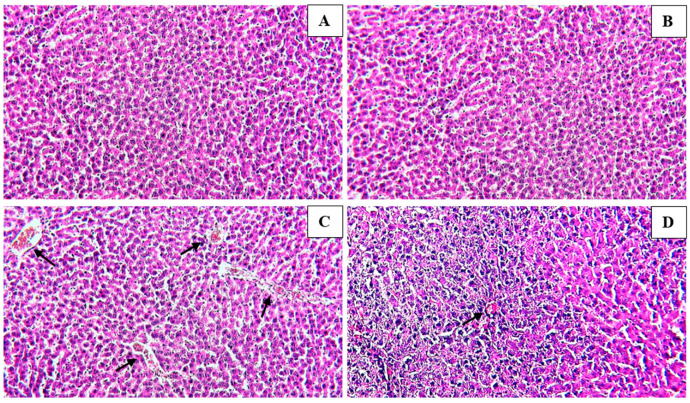
Effect of Paracetamol and/or PC-Ext on liver histopathology photomicrographs of liver sections stained with (H and E). (**A**)—The control group depicted normal histology of hepatocytes. (**B**)—Normal group shows no histopathological alteration and a normal histological structure. (**C**)—Paracetamol-only group treated rats showed significant hepatic injury as indicated by severe congestion and dilatation in the portal vein associated with inflammatory cell infiltration and edema in the periductal tissue surrounding the cystic dilated bile ducts. (**D**)—Paracetamol + *Petroselinum crispum* (200 mg/kg bwt)-treated rats observed maximum improvement with minimal congestion in the portal vein. (**A**–**D**; 20×; Scale bars: 100 μm).

The liver section of the control group revealed normal liver parenchyma, with the central vein clearly seen along with the bile ducts and hepatic arteries (Figure 3A). No histopathological alteration and a normal histological structure were observed in rats administered 200 mg/kg of the extract (Figure 3B). Histopathological changes were seen in the livers of rats given 200 mg·kg^−1^ of Paracetamol; these changes included severe congestion, dilation of the portal vein with infiltration of inflammatory cells, and swelling of the periductal tissue around the cystic, dilated bile ducts (Figure 3C). Histopathological analysis of the liver tissue of rats pre-treated with 200 mg·kg^−1^ of the hydroethanolic extract revealed a maximum degree of protection when compared with the group aided with only 200 mg·kg^−1^ of Paracetamol. Nonetheless, there was congestion and mononuclear cell infiltration, which reached the Paracetamol-treated group to a lesser degree (Figure 3D).

**Figure 4:**
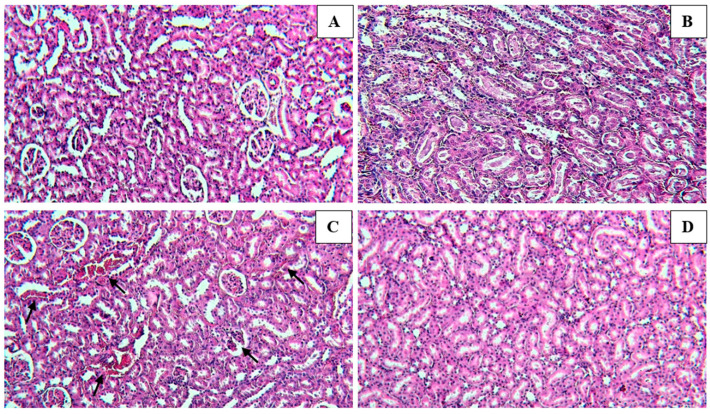
Histopathological changes of kidney sections after being treated with paracetamol and/or *Petroselinum crispum* stained with H & E. (**A**)—The control group revealed well-preserved tubular epithelial cells and the glomerular form of renal cells. (**B**)—The normal group revealed normal renal histology, similar to a normal control group. (**C**)—Paracetamol (200 mg/kg/day, p.o.)-treated rats exhibited massive inflammatory cell infiltration in the deteriorated renal tubules at the cortex, dilatation of the peritubular part, tubular degeneration, and vascular congestion. (**D**)—Paracetamol + PC-Ext (200 mg/kg bwt) treated rats; no histopathological alterations were recorded. (**A**–**D**; 20×; Scale bars: 100 μm).

Microscopically renal tissue in the control group showed well-preserved tubular epithelial cells and normal lobular architecture (Figure 4A). In addition, normal renal histology—similar to a normal control group—was observed microscopically in the normal group (Figure 4B). Paracetamol-treated kidney sections showed abundant massive inflammatory cell infiltration—detected in the degenerated renal tubules at the cortex—and dilatation of the peritubular region, tubular degeneration, and vascular congestion (Figure 4C). Meanwhile, after treatment with both Paracetamol and the PC-Ext, an improvement was recorded in the hepatic tissue. In these specimens, there were no histopathological alterations recorded (Figure 4D).

## 4. Discussion

*Petroselinum crispum*, a plant native to the Mediterranean region, has a long record of medicinal use. It is known for its various pharmaceutical activities due to the bioactive compounds in its leaves, stems, and roots [31].

Paracetamol is a well-known antipyretic and analgesic agent [34] that is safe in therapeutic doses but can produce fatal hepatic necrosis in experimental animals and humans [35]. The ability of *P. crispum* to prevent paracetamol-induced liver damage in rats demonstrated its hepatoprotective activity [36].

Measurements of aspartate and alanine aminotransferase (AST and ALT, respectively) activities can assist in predicting and managing paracetamol poisoning [23]. While these may be promoted by acute Paracetamol use, hepatotoxicity, liver disorders, and rhabdomyolysis are associated with increased aminotransferase activity [37].

In the current study, the significant elevation of biochemical parameters in the rats treated with paracetamol reveals the decay of hepatic function due to the drug’s toxic impacts. Thus, these effects been attributed to damage to the structural integrity of the liver, because these enzymes are unleashed into the circulation after autolytic breakdown or cellular necrosis.

The protections offered by the hydroethanolic extracts of *P. crispum* suggest that they may help treat liver and kidney diseases resulting from Paracetamol overload. The simultaneous administration of Parsley and Paracetamol significantly mitigated the unfavorable effect of Paracetamol on liver and kidney function.

In our study, the plasma level of Paracetamol at admission did not differ between groups (control, normal, and treated with PC+ paracetamol) regarding hepatotoxicity risk.

Our hepatoprotection results align with previous studies by Suhair A. Abdellatief, et al., which showed significant hepatoprotection in vivo and in vitro. In addition, Paracetamol severely decreased the levels of catalase in the liver, which was abolished by treatment with the plant. In fact, *P. crispum* treatment restored catalase activity to levels similar to those found in control mice, reinforcing the plant’s hepatoprotective potential [38].

Treatment with PC-Ext with APAP increases urine volume, possibly due to the considerable diuretic activity of the plant. Parsley prevents proteinuria, anemia, and elevated liver enzymes. In this case, there is competition at the level of the active sites of the eight cytochrome P450 enzymes; the paracetamol is, therefore, transformed into NAPQI at lower levels, so there is no hepatic toxicity [8].

Based on the concentration of the sulfhydryl groups, Coelho et al., estimated the antioxidant protection levels provided by the availability of glutathione to eliminate NAPQI upon APAP overdose [39]. The hepatoprotective influence of Parsley may occur through its antioxidant potential, the decrease in lipid peroxidation, and the intensification of the antioxidant defense system [11].

Previous phytochemical analysis of Parsley revealed the presence of certain compounds such as flavonoids [40], carotenoids [41], ascorbic acid [42], and tocopherol [43]. These components of fresh Parsley leaves eliminate the superoxide anion in vitro and the hydroxyl radical, in addition to protecting against membrane oxidation induced by ascorbic acid [44]. Stimulating the production of glutathione synthesis and thus strengthening the cellular antioxidant defense [45]. Therefore, Parsley could be an essential therapeutic tool to combat diseases related to oxidative stress.

As mentioned in the introduction, the metabolism of high-dose APAP in the liver relies on CYP450 isoenzymes, which are liable for the oxidation of the drug and the formation of the reactive metabolite NAPQI [20]. Therefore, heightened CYP2E1 mRNA expression may contribute to APAP-induced hepatotoxicity [5]. Hence, APAP may decrease CYP2E1 activity in vivo to counter-regulate the appearance of toxic NAPQI [46].

Parsley impacts cytochrome P450 isoforms and may inhibit or activate cytochrome, depending on the CYP isoform and the dose of extract utilized [15].

The present investigation indicated that Parsley extract carried almost normal serum protein and albumin levels, most likely due to the preservation of liver function and the reduction of albuminuria.

In closing, the outcomes of this study revealed the high efficacy of the crude hydroethanolic extracts of *P. crispum* in functional foods and their effectiveness in treating different diseases—among which, liver and kidney disease are the most important. However, although the use of *P. crispum* extract is safe for humans, elaborate clinical tests have yet to be carried out to evaluate this plant’s potential hepatotoxicity after administering heightened doses to test animals and healthy human volunteers.

## 5. Conclusions

In conclusion, the findings of this study highlight the protective potential of the ethanolic extract of *Petroselinum crispum* against Paracetamol-induced renal, hepatic, and hematological toxicity in rats. Additionally, it demonstrated the ability to mitigate the histopathological damage observed in the liver and kidneys. These outcomes underscore the likelihood that Parsley’s antioxidant and free radical-scavenging attributes play a pivotal role in its robust hepatoprotective and nephroprotective effects. This research may have significant implications for human health—particularly in managing liver and kidney conditions and addressing proteinuria resulting from Paracetamol overdose. Further research is warranted to pinpoint the precise phytoconstituents responsible for these hepatorenal protective effects, opening avenues for more comprehensive exploration in future studies.

## 6. Future Research Directions

The incorporation of tissue samples in proteomics studies stands as a promising avenue for our future research endeavors. This strategic approach will facilitate a profound exploration of the mechanisms responsible for the protective attributes of Parsley extract, enabling a more holistic grasp of its influence on cellular and molecular processes. Our unwavering commitment to introducing tissue samples into proteomics studies underscores our dedication to the advancement of our research, with the ultimate goal of enriching our comprehension of the advantageous effects of Parsley extract. This innovative approach holds the potential to significantly augment the scientific community’s knowledge on this subject matter. Furthermore, it may bear implications for the development of future therapeutic interventions, thus offering a valuable contribution to the broader field of health and wellness.

## Figures and Tables

**Table 1:** Serum biochemical parameters at the 4th week post-treatment with PC-Ext in Paracetamol-intoxicated rats.

Blood Variables	Control	Normal Group	APAP-Control	APAP + PC-Ext
ALT (U/L)	89.5 ± 3.54	82 ± 2.83	143.11 ± 0.34 ^a^***	111.5 ± 12.02 ^a^***^,b^***
AST (U/L)	223 ± 31.11	231 ± 4.35	282 ± 7.88 ^a^***	232 ± 1.08 ^b^***
ALP (UI/L)	238.5 ± 3.1	245.5 ± 9.09 ^a^***	337.5 ± 4.09 ^a^***	262.5 ± 7.9 ^a^***^,b^***
Albumin (g/L)	43.15 ± 3.75	46.7 ± 3.82	34.25 ± 0.35	44.15 ± 0.45
Creatinine (mg/L)	4.1 ± 0.13	4.45 ± 0.52	5.57 ± 0.34	4.04 ± 0.25
BUN (g/L)	0.24 ± 0.06	0.36 ± 0.04	0.49 ± 0.12	0.38 ± 0.00
Uric acid (mg/L)	18.93 ± 11.78	16.1 ± 7.09	13.26 ± 0.62	17.82 ± 0.40
Protein (g/L)	66.67 ± 2.35	67.32 ± 7.28	56.5 ± 3.02	65.30 ± 5.38
LDH (U/L)	107.15 ± 1.48	91 ± 5.6 ^a^*	265.10 ± 4.76 ^a^***	130.35 ± 8.13 ^a^***^,b^***
Triglycerides (g/L)	0.4 ± 0.14	1.01 ± 0.28	2.13 ± 2.88	1.04 ± 0.25

Each value represents mean ± S.E.M. * *p* < 0.05, *** *p* < 0.001, ^a^ as compared with control group; ^b^ comparison between the Paracetamol group and the remaining groups. Statistical analysis by Two-way ANOVA followed by Dunnett’s multiple comparisons tests. AST: Aspartate aminotransferase, ALT: Alanine aminotransferase, ALP: Alkaline phosphatase, BUN: Blood urea nitrogen, LDH: Lactic acid dehydrogenase.

**Table 2:** Effect of Paracetamol overdose and PC-Ext on hematological parameters.

Parameters		Control	Normal Group	APAP-Control	APAP + PC-Ext
White blood cell (mm^3^)		8230.00 ± 3.1	8143.7 ± 3.01 ^a^***	13020 ± 6.11 ^a^***	7261.5 ± 2.43 ^a^***^,b^***
Red blood cell (M/mm^3^)		8.56 ± 0.66	8.15 ± 0.77	5.02 ± 0.34	6.65 ± 0.98
Hemoglobin (g/dL)		14.1 ± 0.4	14.15 ± 0.92	8.98± 1.0 ^a^*	10.9 ± 1.13
Hematocrit (%)		49.3 ± 1.2	46.6 ± 4.95	36.45 ± 4.22 ^a^***	44.85 ± 5.02 ^b^**
VGM (u^3^)		57.6 ± 2.06	57.2 ± 0.71	48.62 ± 1.23 ^a^***	55.45 ± 0.64 ^b^*
TCMH (pg)		16.5 ± 1.1	17.4 ± 0.57	15.6± 0.2	16.4 ± 0.71
CCMH (g/dL)		34.6 ± 0.42	33.4 ± 1.27	28.6 ± 0.41 ^a^*	30.65 ± 0.92
Platelets (10^3^/mm^3^)		841 ± 4.02	818.5 ± 2.15 ^a^***	962 ± 10.27 ^a^***	722 ± 3.76 ^a^***^,b^***
VPM (FL)		7.6 ± 0.3	8 ± 0.42	6.02 ± 0.21	7.8 ± 0.42
Differential count (%)	Neutrophils (%)	24.5 ± 0.2	23.7 ± 0.19	30.1 ± 2.90 ^a^*	25 ± 1.27 ^b^*
	Eosinophils (%)	0.00	0.00	0.00	0.00
	Basophils (%)	0.00	0.00	2.0 ± 0.7	1 ± 1.41
	Lymphocytes (%)	53.5 ± 1.41	54 ± 1.04	60 ± 2.13 ^a^**	45 ± 9.90 ^a^***^,b^***
	Monocytes (%)	2 ± 0.1	3 ± 1.41	3.5 ± 0.1	3 ± 1.41
	Granulocytes (%)	0.0	0.1 ± 0.00	0.7 ± 0.02	0.05 ± 0.07

Each value represents mean ± S.E.M. * *p* < 0.05, ** *p* < 0.01, *** *p* < 0.001, ^a^ as compared with the control group; ^b^ comparison between the Paracetamol group and the remaining groups. Statistical analysis by Two-way ANOVA followed by Dunnett’s multiple comparisons tests. VGM: Mean corpuscle, TCMH: Mean corpuscle hemoglobin content, CCMH: Mean corpuscle hemoglobin concentration, VPM: Mean platelet volume, PC: *Petroselinum crispum*, APAP: Paracetamol.

**Table 3:** Effect of interventions on urinary volume and pH.

Interventions mg/kg BWT		Control	Normal Group	APAP-Control	APAP + PC-Ext
Urine volume (mL/24 h)	D0	6.2 ± 0.41	7.25 ± 1.8	6 ± 1.3	6.10 ± 0.42
	D7	7.4 ± 0.19	10.3 ± 0.1 *	7.5 ± 0.04	7 ± 0.23
	D15	6.3 ± 0.5	16.5 ± 0.5 ***	8 ± 1.2	10.72 ± 0.7 **
F/*p* values			0.014/0.924	0.121/0.773	0.067/0.832
Urine pH	D0	6.53 ± 0.55	6.61 ± 0.82	6.54 ± 1.04	6.67 ± 0.15
	D7	6.44 ± 0.64	6.57 ± 1.2	6.75 ± 0.58	6.38 ± 0.72
	D15	6.45 ± 1.49	6.89 ± 0.63	7.53 ± 0.35	6.04 ± 0.56
F/*p* values			0.622/0.421	0.962/0.124	0.899/0.205

Each value represents mean ± S.E.M. * *p* < 0.05, ** *p* < 0.01, *** *p* < 0.001 as compared with control group. Statistical analysis by Two-way ANOVA followed by Dunnett’s multiple comparisons tests.

## Data Availability

Not applicable.
